# Interleukin-1 receptor antagonist treatment revealed active hepatitis B infection in a boy with PAPA syndrome

**DOI:** 10.1186/1546-0096-13-S1-P11

**Published:** 2015-09-28

**Authors:** V Selmanovic, F DeBenedeti, A Omercahić-Dizdarevic, E Kovac-Vidakovic, S Mehanic, A Cengic

**Affiliations:** 1 Children's Hospital University Clinical Center Sarajevo, Department for Allergology, Rheumatology and Clinical Immunology, Sarajevo, Bosnia and Herzegovina; 2 IRCCS Ospedale Pediatrico Bambino Gesù, Division of Rheumatology, Roma, Italy; 3 Children's Hospital University Clinical Center Sarajevo, Department for Gastroenterology and Hepatology, Sarajevo, Bosnia And Herzegovina; 4 University Clinical Center Sarajevo, Hospital for infectious diseases, Sarajevo, Bosnia And Herzegovina

## Background

PAPA syndrome (pyogenic sterile arthritis, pyoderma gangrenosum and acne) is a rare autosomal-dominant autoinflammatory disease caused by mutations in PSTPIP1gene. Typically presents with recurrent sterile, erosive arthritis in childhood, occurring spontaneously or after minor trauma, occasionally resulting in significant joint destruction. By puberty, joint symptoms tend to subside and cutaneous symptoms increase. Cutaneous manifestations include pathergy, frequently with abscesses at the sites of injections, severe cystic acne, and recurrent nonhealing sterile ulcers, often diagnosed as PG.

## Objective

To report a case of hepatitis B infection revealed with interleukin-1 receptor antagonist for PAPA syndrome.

## Patient and method

16,5y boy with PAPA syndrome presenting at age 2 with pyogenic sterile arthritis requiring multiple surgeries (shoulders, elbows, knees, ankles, wrists). He was treated as JIA for 14y (NSAID, steroids (11 years, had growth retardation due to steroids), immunosupressants (metotrexate) and for short time biologicals (infliximab, adalimumab). Several years ago developed severe acne and nonhealing skin ulcers. In august 2013, the boy was referred to our Department and completely reevaluated. He had raised inflammatory markers, microcytic anaemia, normal transaminases. Skin biopsy: pyoderma gangrenosum. Kidney biopsy excluded amyloidosis. Started with adalimumab for 1 year. Joint disease was under control, but not skin disease. Clinical diagnosis of PAPA syndrome was confimed by genetic analysis in March 2014. He was heterozygous for the substitution 748G>C in exon 11 that predicts the E250Q aminoacid substitution. When available, adalimumab was switched to anakinra in November 2014 (after washout period of 8 weeks). One month later, the patient showed raised transaminases (10 times above upper limit of normal). Active hepatitis B infection was proved by PCR. His mother had hepatitis B and boy had several surgeries with blood transfusions. How did he harbour the infection? Anakinra was stopped. On symptomatic therapy, transaminasemia soon normalised, but PAPA worsened. At present, he is on antiviral therapy and the issue if and when anakinra should be restarted.

## Conclusion

We reported a boy with genetically confirmed PAPA syndrome. Treatment with adalimumab controlled joint but not skin disease. Interleukin-1 receptor antagonist was associated with activation of previously unknown hepatitis B infection, requiring antiviral therapy and withdrawal of treatment with anakinra. This case raises the question of the possible role of anakinra in activating the viral infection and the usefullness routine srceening for hepatitis B prior biological.

## Consent to publish

Written informated consent for publication of their clinical details was obtained from the patient/parent/guardian/relative of the patient.

## References

[B1] Dierselhuis MP Frenkel J Wulfraat NM Boelens JJ Anakinra for Flares of Pyogenic arthritis in PAPA Syndrome Rheumatology 2005 44 3 British Society for Rheumatology 10.1093/rheumatology/keh479 15637033

[B2] Brener M Ruzicka T Plewig G Thomas P Herzer P Targeted treatment of pyoderma gangrenosum in PAPA (pyogenic arthritis, pyoderma gangrenosum and acne) syndrome with the recombinant human interleukin -1 receptor antagonist anakinra British Journal of Dermatology 2009 161 1199 1201 10.1111/j.1365-2133.2009.09404.x 19673875

[B3] Caorsi R Insalaco A Marotto D Frenkel J Martini A De Benedetti F Gattorno Mpediatric Rheumatology 2013 11 2 P228

[B4] Caorsi R Federici S Gattorno M Biologic drugs and autoinflammatory syndromes Autoimmunity Reviews 2012 12 81 86 10.1016/j.autrev.2012.07.027 22884553

[B5] Touitou I Pyogenic artheritis - pyoderma gangrenosum - acne. Th portal for Rare disease and orphan drugs, october 2006 dostupno na : http://orpha.net/consor/cgi-bin/OC_Exp.php?Lng=EN&Expert=69126

[B6] Demidowich AP Freeman AF Kuhns DB Aksentijevich I Gallin JI Turner ML Kastner DL Holland SM Genotype, phenotype and Clinical Course in Five Patients With PAPA Syndrome (pyogenic sterile arthritis, pyoderma gangrenosum and Acne) Arthritis 6 Rheumatism 2012 64 6 2022 2027 10.1002/art.34332 PMC3737487 22161697

